# Exceptionally stable membrane lipid composition of the marine facultative anaerobe and piezotolerant ‘*Labilibaculum euxinus*’ under variable pressure and nutrients

**DOI:** 10.1093/femsec/fiag012

**Published:** 2026-02-18

**Authors:** Anandi Tamby, Diana X Sahonero-Canavesi, Nicole J Bale, Laura Villanueva

**Affiliations:** Department of Marine Microbiology and Biogeochemistry (MMB), NIOZ Royal Netherlands Institute for Sea Research, 1797SZ't Horntje, The Netherlands; Department of Marine Microbiology and Biogeochemistry (MMB), NIOZ Royal Netherlands Institute for Sea Research, 1797SZ't Horntje, The Netherlands; Department of Marine Microbiology and Biogeochemistry (MMB), NIOZ Royal Netherlands Institute for Sea Research, 1797SZ't Horntje, The Netherlands; Department of Marine Microbiology and Biogeochemistry (MMB), NIOZ Royal Netherlands Institute for Sea Research, 1797SZ't Horntje, The Netherlands; Department of Biology, Faculty of Sciences, Utrecht University, 3584CH Utrecht, The Netherlands

**Keywords:** high hydrostatic pressure, membrane lipids, marine microorganism, piezotolerance, phosphate, ‘*Labilibaculum euxinus*’

## Abstract

The lipid bilayer is a dynamic barrier that plays a critical role as a frontier between cellular elements and the environment. The marine environment presents a unique set of conditions, including variations in nutrient availability, temperature, and high hydrostatic pressure (HHP). To survive these conditions, deep-sea microbes have developed adaptive strategies to preserve the integrity of their lipid membranes, particularly in response to HHP. Here, we assessed these adaptations by determining changes in the membrane lipids of a piezotolerant bacterium, ‘*Labilibaculum euxinus*’ from the phylum *Bacteroidota* (family *Marinifilaceae*) isolated from 2000 meters depth in the Black Sea, under different hydrostatic pressures and phosphate concentrations. Lipid analysis of ‘*L. euxinus*’ grown in a medium replete in phosphate revealed the presence of lipids with nonphosphate containing amino acid headgroups, such as ornithine lipids, flavolipins, and capnine lipids, typically associated with adaptation to phosphate limitation. Microscopy analysis revealed cell elongation under HHP, suggesting cell adaptation. Despite this morphological change, the distribution of membrane lipids remained stable in terms of polar headgroups. Nevertheless, HHP affected the unsaturation of the fatty acyl chain and the relative abundance of cardiolipins. Our study showcases the adaptability of certain extremophiles, particularly piezotolerant organisms.

## Teaser

This study reveals how deep-sea bacteria adapt their membrane lipids to survive high pressure and nutrient limitations, highlighting the remarkable resilience of microbes in extreme marine environments..

## Introduction

The microbial membrane is an essential component of the cell, ensuring vital functions such as cell communication, maintenance of cell integrity, nutrient acquisition and waste removal (Šajbidor 1997, Guan et al. 2017). Membranes are composed of proteins and lipids. In bacteria, those membrane lipids are generally composed of a polar headgroup attached to two hydrophobic fatty acyl chains (known also as core lipids) via ester bonds to glycerol-3-phosphate. The fatty acyl chains can have various lengths, degrees of unsaturation and methylation (Siliakus et al. 2017). In their intact polar lipid (IPL) form, both the core lipids and the polar headgroups determine many properties of the phospholipid and, consequently, the structure and function of the membrane (Maltseva et al. 2022, Yu et al. 2024). Specifically, membrane lipid headgroups influence membrane fluidity, asymmetry, and functionality, and serve crucial roles in cell recognition, signaling pathways, and enzymatic reactions (Seu et al. 2006, Frolov et al. 2011, Cheng and Smith 2019). Membrane lipid headgroups can be classified in two groups: phosphate-containing such as phosphatidylcholine (PC), phosphatidylethanolamine (PE), phosphatidylserine, among others, and nonphosphate containing, such as glycolipids, amino acid-containing lipids (i.e. amino lipids), such as ornithine lipids, capnine lipids, flavolipins, and glycine lipids (Geiger et al. 2010) (Supplementary Fig. 1).

Microorganisms in the marine environment, especially those in the deep sea, have developed strategies to adapt to changes in nutrient availability, temperature, and high hydrostatic pressure (HHP), to preserve the integrity of their lipid membranes (DeLong and Yayanos 1986, Wirsen et al. 1986, among others). Regarding hydrostatic pressure response, microorganisms are classified as piezophiles if they depend on elevated pressure for growth, and piezotolerant if they can withstand such conditions without being pressure dependent (Abe and Horikoshi 2001).

Changes in environmental conditions can alter the fluidity and stability of the lipid membrane and affect membrane permeability (Beney and Gervais 2001). Among these factors, HHP, a constant parameter of the deep-sea, has been seen to affect membrane lipid fatty acid saturation and lipid packing in general (Bartlett 2002). Homeoviscous adaptation of the cell membrane is also a vital feature for microorganisms in general and marine ones in particular, enabling them to adjust the length and saturation of membrane lipid fatty acids, ensuring optimal fluidity under challenging environmental conditions (Zhang and Rock 2008, Ernst et al. 2016). To maintain membrane fluidity through homeoviscous adaptation,, microorganisms have been seen to increase the relative abundance of phospholipids with unsaturated and branched fatty acids with decreasing temperature and increasing pressure (Bartlett 1999). However, the impact of HHP on IPLs diversity remains largely unknown.

Other physiological parameters such as redox conditions, pH and nutrient concentration have been identified to affect membrane lipid composition, and thus influencing cellular physiology (Siliakus et al. 2017, Vences-Guzmán et al. 2011, Itri et al. 2014). Phosphate availability notably also impacts the membrane lipid composition. Studies observing changes in the membrane lipid composition in marine phytoplankton and in heterotrophic bacteria in culture, defined a low phosphate culture medium as containing <0.5–1 µM phosphate, which induced the production of nonphosphorous (non-P) lipids such as ornithine lipids, sulfolipids, or glycolipids to maintain membrane integrity and function (Martin et al. 2010, Vences-Guzmán et al. 2011, Carini et al. 2015, Sebastián et al. 2016, Hunter et al. 2018, Lejeune et al. 2021, Westermann et al. 2023). In addition, marine field-based studies have observed a dominance of non-P lipids at phosphate concentrations of <100 nM phosphate (van Mooy et al. 2006, 2009, Schubotz et al. 2018).

Marine microorganisms are faced with multiple environmental and metabolic stresses that have been reported to affect their membranes, nevertheless the synergistic effect that multiple factors can have on the microbial lipid membrane remains unexplored, particularly when one of them is HHP. Synergistic impact of multiple environmental challenges could affect membrane lipid composition, fluidity and integrity, and understanding this interplay can shed light on adaptive strategies used by microorganisms in marine environments.

The above-mentioned changes in the cell lipid composition can also lead to alterations in cell shape, with consequences for envelope integrity and cell physiology (Rowlett et al. 2017). Notably, under nutrient limitation bacteria might change their morphology to optimize the surface area and nutrient absorption. HHP has been observed to impair functions such as DNA replication or protein folding, leading to cell division impairment (Bartlett 2002). However, little is known about the effect of environmental factors on cell morphology and membrane stability due to the changes in the membrane lipid composition. Here, we determined the effects (individual and combined) of HHP and nutrient reduction (i.e. phosphate), both in the membrane lipids and morphology, on a facultative anaerobic bacterial strain, ‘*Labilibaculum euxinus*’ of the family *Marinicifiliceae*, isolated from the euxinic waters of the Black Sea at 2000 m depth (Yadav et al. 2020). Members of the family *Marinifilaceae* have been reported to make up 1%–7% of the total microbial community in the Black Sea water column (Yadav et al. 2020, 2024). In laboratory enrichments with complex carbon sources, their relative abundance increased, suggesting that ‘*Labilibaculum*’ and other members of the *Fibrobacteres–Chlorobi–Bacteroidetes* (FCB) group may act as ‘seed microbes’ under occasional copiotrophic (nutrient-rich) conditions in the Black Sea, where they likely play a key role in degrading organic matter (Yadav et al. 2024). The ‘*L. euxinus*’ strain used in the current study was described as piezotolerant, growing from 0.1 MPa (atmospheric pressure) to 40 MPa (4000 m depth), and synthesizing a high percentage of nonphosphate based amino lipids (Yadav et al. 2020). Members of the phylum *Bacteriodota* generally have a rather characteristic IPL composition, being the major membrane lipids PE, phosphatidylglycerol, cardiolipin, and amino lipids (mostly ornithine lipids) (Sohlenkamp and Geiger 2015).

In this study, we determined the effect of nutrient (i.e. phosphate) reduction, as well as HHP, in both the lipid composition and cell morphology of *L. euxinus* to draw conclusions regarding microbial adaptation to multiple stresses. In addition, we also investigated the amino lipid genomic biosynthetic capacity of this strain, which can help elucidate the biosynthetic steps leading to the synthesis of specific amino lipids in bacteria.

## Materials and methods

‘*Labilibaculum euxinus*’ A4^T^, a facultative anaerobic piezotolerant microorganism (0.1 to 50 MPa) of the phylum *Bacteroidota* family *Marinifilaceae*, was previously isolated from the Black Sea water column at 2,000 m depth (Yadav et al. 2020). In our previous study (Yadav et al. 2020), ‘*L. euxinus*’ was grown for lipid analysis in rich media containing yeast extract and tryptone and incubated at both atmospheric (0.1 MPa) and at HHP (20 MPa). In this study, we assessed the growth of ‘*L. euxinus*’ in a defined medium with pyruvate as the only carbon source and with different phosphate concentrations to evaluate differences in the lipid composition under different conditions. Two phosphate concentrations were chosen, 1.4 mM, which corresponded to the phosphate concentration in rich medium in which ‘*L. euxinus*’ was originally isolated, hereafter defined as ‘replete’, and 0.35 mM, which was the lowest phosphate concentration reached that could still sustain growth at 20 MPa as tested in the current study (data not shown), hereafter referred to as ‘reduced’.

### Media preparation and growth of ‘*L. euxinus*’

‘*Labilibaculum euxinus*’ was originally grown anaerobically in seawater medium (SWM; rich medium) at optimum temperature of 28°C. The optimum growth temperature of ‘*L. euxinus*’ is 20°C−28°C, and the range of growth is between 4°C and 35°C (Yadav et al. 2020). The *in situ* temperature at 2000 m depth, where ‘*L. euxinus*’ was isolated from, is very stable throughout the year and approximately of 8°C (Suominen et al. 2021; among others). The amount of total biomass obtained at 8°C−10°C was too low to perform lipid analysis (data not shown). Hence, our choice of the highest temperature of growth (28°C) which is still within the range of growth temperature, ensuring highest biomass yield.

‘*Labilibaculum euxinus*’ was transferred from the rich SWM to a defined medium containing KH_2_PO_4_ (0.20 g/L), NH_4_Cl (0.25 g/L), NaCl (20 g/L), MgCl_2_×6H_2_O (3 g/L), KCl (0.5 g/L), CaCl_2_x2H_2_O (0.15 g/L), MOPS (8.3 g/L), resazurin (1 mg/mL), and pyruvate (2 g/L), at pH 7. Medium was boiled to remove oxygen and subsequently flushed for 45 min with N_2_. The media was then transferred to pressure bottles of 250 ml to a maximum volume of 120 ml of medium and further flushed for 30 min with N_2_ in the medium, and 20 min in the headspace. The bottles were autoclaved at 121°C for 20 min and kept at 28°C until inoculation. Growth in this medium contained 1.4 mM phosphate as KH_2_PO_4_ (0.20 g/L) defined here as ‘replete’ phosphate conditions, which was equivalent to the phosphate concentration present in the rich SWM where ‘*L. euxinus*’ was originally grown. Once ‘*L. euxinus*’ was acclimated to the defined medium with ‘replete’ phosphate conditions (at least 100 transfers in the defined medium), the culture (1/100 dilution) was transferred to media with lower phosphate concentrations. Phosphate concentration was estimated as described in Bale et al. (2013). Below 0.35 mM phosphate, the culture was either not growing or taking several weeks in latency period but never reaching equivalent OD_600nm_ to the culture grown in ‘replete’ phosphate concentration at 20 MPa. To this end, concentration of 0.35 mM was the minimum concentration of phosphate leading to growth at 20 MPa, and it is referred here as ‘reduced’ phosphate concentration (i.e. KH_2_PO_4_, 0.05 g/L).

For the growth curves of ‘*L. euxinus*’ at atmospheric pressure (0.1 MPa), the optical density at 600 nm (OD_600nm_) was assessed twice per day with a 9-h interval by measuring in both the replete (1.4 mM) and reduced phosphate (0.35 mM) concentrations. For incubations under HHP, a high-pressure assembly was used (Yadav et al. 2020). Prior to incubation, liners, pistons, and vessels were assembled and then autoclaved at 121°C for 20 min. The vessels were then transferred to an anaerobic glovebox (max O_2_ concentration of 5 ppm) where they were left overnight. The vessels were then mounted on the assemblies and filled with anoxic media inoculated with ‘*L. euxinus*’ (35 ml per vessel). For both atmospheric and HHP incubations, the inoculation was made by using a stationary culture after 5 days diluted 1:100 in fresh medium. The assemblies were then pressurized to 20 MPa and left in an incubator at 28°C without shaking for 120 h, until the OD_600nm_ was expected to be 0.1 based on previous test experiments. Depressurization was performed slowly inside an anaerobic glove bag, before proceeding with the opening of the assembly for subsampling to measure the OD_600nm_. The rest of the biomass was harvested by filtration using muffled glass filter fibers 0.3 µm diameter pore size (GF75, Advantec, Toyo Roshi Kaisha, Ltd).

### Lipid extraction and analysis

IPLs were extracted from freeze-dried ‘*L. euxinus*’ biomass twice in an ultrasonic bath for 10 min in solution of methanol:dichloromethane:phosphate (2:1:0.8, v:v). The supernatants were then combined and phase-separated by adding dichloromethane and phosphate buffer to a final ratio of 1:1:0.9 (v:v). The organic phase was collected, and the aqueous phase was extracted twice with dichloromethane. The biomass residue was then re-extracted following the same procedure but with a mixture of MeOH:DCM:TCA (aqueous trichloroacetic acid solution; pH 3; 2:1:0.8, v:v). Finally, all organic extracts were combined and dried under N_2_. For analysis, the extracts were redissolved in MeOH:DCM (9:1, v:v) which contained an internal standard, a deuterated betaine lipid (1,2-dipalmitoyl-*sn*-glycero-3-O-4′-[N, N, N-trimethyl(d9)]-homoserine; Avanti Lipids), before filtration through 0.45 mm regenerated cellulose syringe filters (4 mm diameter; Grace Alltech).

The extracts were analyzed by ultra-high performance liquid chromatography coupled with high-resolution mass spectrometry (UHPLC-HRMS) according to ​Bale et al. (2021). To conduct IPL analysis, we used an Agilent 1290 Infinity I UHPLC equipped with thermostatted autoinjector and column oven, coupled to a Q Exactive Orbitrap MS with an Ion Max source and heated electrospray ionization (HESI) probe (Thermo Fisher Scientific). Separation was achieved on an Acquity BEH C18 column (Waters, 2.1 × 150 mm, 1.7 mm) maintained at 30°C. We used an eluent composition of (A) methanol/water/formic acid/14.8 M NH_3_aq [85:15:0.12:0.04 (v:v)] and (B) isopropyl alcohol/methanol/formic acid/14.8 M NH_3_aq [50:50:0.12:0.04 (v:v)]. The elution program was: 95% A for 3 min, followed by a linear gradient to 40% A at 12 min and then to 0% A at 50 min, this was maintained until 80 min. The flow rate was 0.2 ml min^−1^. Positive ion HESI settings were: capillary temperature, 300°C; sheath gas (N_2_) pressure, 40 arbitrary units (AU); auxiliary gas (N_2_) pressure, 10 AU; spray voltage, 4.5 kV; probe heater temperature, 50°C; and S-lens 70 V. Lipids were detected using a mass range of m/z 350–2000 and MS^2^ spectra were obtained via data-dependent acquisition, where the top 10 abundant ions per MS^1^ scan were selected for fragmentation. The Q Exactive was calibrated within a mass accuracy range of 1 ppm using the Thermo Scientific Pierce LTQ Velos ESI Positive Ion Calibration Solution. During analysis, dynamic exclusion was used to temporarily exclude masses (for 6 s) to allow selection of less abundant ions for MS/MS. Stepped normalized collision energy of 15, 22.5, and 30 was used for fragmentation. Identification of the IPL composition of ‘*L. euxinus*’ membrane was done according to Yadav et al. (2020). IPLs were examined in terms of the peak area of the summed mass chromatograms of the protonated, ammoniated and sodiated ions ([M+H]^+^, [M+NH_4_]^+^, [M+Na]^+^) within 3 ppm relative mass tolerance. It should be noted that IPLs have varying degrees of ionization efficiency and hence the peak areas of different IPLs do not necessarily reflect their actual relative abundance. But this method allows for comparison of samples analyzed together. Statistical analysis of the differences between the IPL classes under different conditions were determined by using a two-way ANOVA corrected for multiple comparisons using Tukey’s multiple comparison test with a confidence interval of 95%.

### Microscopic analyses

For microscopic analysis, 1 ml of growing culture was sampled and collected by centrifugation (10 min, 13 000 r/m). The supernatant was then discarded, and the cells were re-suspended in 50 µL of growth medium. The cells were then transferred on an agarose pad (0.5% agarose) as described in Sahonero-Canavesi et al. (2022). The cells were visualized using a Zeiss Axio Imager M2 equipped with Axiocam 705 color and Axiocam 705 mono. Data of cell characteristics (length, width, area and circularity) were gathered using ImageJ and MicrobeJ 5.13p. We investigated morphological changes with a population of *n* = 77 cells for each condition. Each experimental condition was analyzed in triplicate through this method. Statistical analysis was performed using GraphPad Prism (10.1.2) software. Differences between conditions were assessed using ordinary one-way ANOVA test, with a significance level set at *P* < .05. Here, length is defined as the medial axis of the particle in µm, width as the measurement of the cell from side to side along the medial axis of the particle in µm, area as the calculated square µm^2^ occupied by the cells and circularity defined as 4π × area/perimeter^2^ (with values ranging from 0 to 1, being 1 a perfect circle/spherical shape).

### Genomic analyses

#### DNA extraction

Biomass was preserved at −80°C. Prior to extraction, filters were thawed on ice and cut into pieces with sterile scalpels. A cetyltrimethylammonium bromide (CTAB) buffer composed of 100 mM TrisHCl adjusted to pH 8, 20 mM EDTA, 1.4 M NaCl, and 0.05 M CTAB was prepared. Prior to extraction, Lysosyme and RNAse were then added to the buffer to a final concentrations of 0.8 mg mL^−1^ and 5 µg mL^−1^. Filter pieces were covered with CTAB buffer containing lysozyme and RNAse and incubated at 37°C for 60 min, after which proteinase K was added to a final concentration of 0.2 mg mL^−1^. SDS 10% was added to a ratio of 1:20. The tubes were then incubated for 60°C for 1 h, with gentle mixing every 10 min. A mixture of phenol:chloroform:isoamyl alcohol (25:24:1) was then added to a ratio of 1:1. Tubes were centrifuged at 1500 × *g* for 5 min. The aqueous phase was recovered into a new tube. A mixture of chloroform:isoamyl alcohol (24:1) was added and mixed to the aqueous phase, followed by centrifugation (1500 × *g* for 5 min). The aqueous phase was recovered and washed with chloroform, followed by centrifugation (1500 × *g* 5 min). Glycogen (5 mg/mL) was then added to a ratio of 1:100, 3 M sodium acetate pH 5.5 to a ratio of 1:10 and cold ethanol to a ratio of 2.5:1. The solution was then precipitated for 1 h at −20°C, and centrifuged for 20 s at 13 000 r/m, 4°C. Ethanol was then discarded, and pellet washed in cold 70% ethanol five times, after which they were resuspended with 50 ml of TE 1x pH 8.0 buffer by pipetting very gently. The extract was stored at 37°C for 30 min before storing at −80°C.

#### Genome sequencing and analysis

DNA extract was sequenced by means of the MinION Mk1C equipped with a cell type FLO-MIN106 with the kit type SQK-LSK110. The runs lasted 72 h, with active channel selection, 1.5 h of pore scan frequency and minimum read length of 1000 bp. The software versions were MinKNOW 22.08.9, Bream 7.2.9, Configuration 5.2.8, MinKnow Core 5.2.8.

The basecalling was done with Guppy version 6.4.2 and the alignment with Flye version 2.9.3-b1797, in metagenomic mode. Homology search of amino lipid biosynthetic genes in the genome of ‘*L. euxinus* A4^T^’ were performed by protein blast with release NCBI Blast+ (2.11.0). Accession numbers of the query sequences were: GlsA, O-acyltransferase (BT_3458*, Bacteroides thetaiotaomicron* VPI-5482, accession number NCBI AAO78564.1, Phospholipid/glycerol acyltransferase); GlsB, N-acyltranferase (BT_3459, *Bacteroides thetaiotaomicron* VPI-5482, accession number (AAO78565.1, annotated as a hemolysin A) (Lynch et al. 2019), OlsA *Sinorhizobium meliloti* (AEH77326.1), OlsB *S. meliloti* (ATA97800.1), OlsF *Serratia proteamaculans* (QQX52868.1) (Vences-Guzmán et al. 2015); CapA (Uniprot C7M8J5, NCBI accession ACU93374.1 *Capnocytophaga ochracea;* Liu et al. 2022), CapB (C7M8J6; ACU93375.1), and CapC (A0A3R5XUE6, QAR30703.1, *Ornithobacterium rhinotracheale*).

## Results

### Growth of ‘*L. euxinus*’ in defined media with different phosphate concentrations and at atmospheric pressure

**Figure 1:**
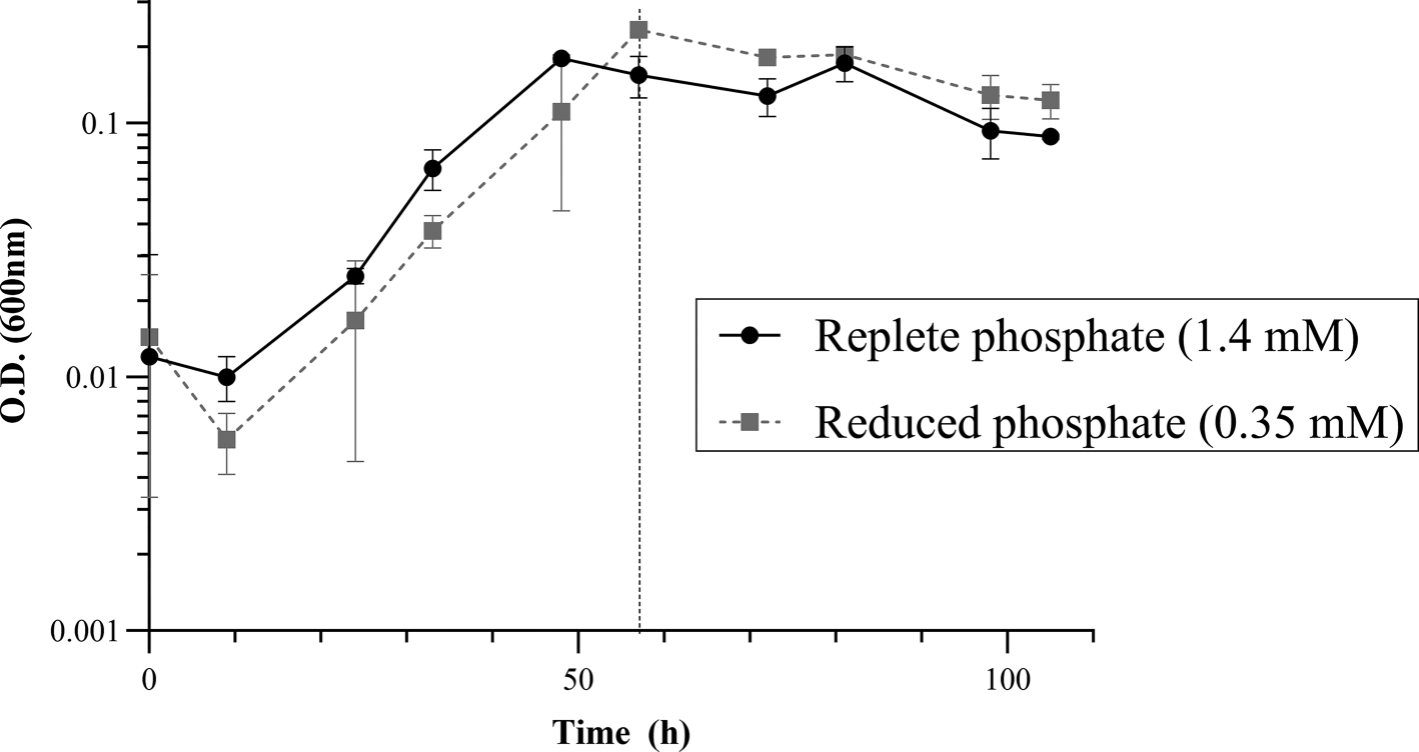
Growth curve of ‘*L. euxinus*’ A4^T^ in media with replete and reduced phosphate. The dotted line indicated *t* = 57 h, which was used in subsequent culture experiments as harvesting time during late exponential/early stationary phase. Note that the optical density, OD_600nm_ is low, while in the rich medium used by Yadav et al. (2020), the OD_600nm_ was ~0.8–1 in stationary phase of growth.

‘*Labilibaculum euxinus*’ was transferred from a rich medium (Yadav et al. 2020) to a defined medium with pyruvate as a carbon source, and replete phosphate concentration (1.4 mM), and then subtransferred at least 100 times before the strains were considered as acclimated to the defined medium. Once the strain was acclimated to the defined medium with the replete phosphate concentration, and grown to saturation for 5 days, a 1:100 dilution of this culture was performed to follow up the growth curve under atmospheric pressure (0.1 MPa) both in defined medium with replete phosphate, and in defined medium with reduced phosphate concentration (0.35 mM), which was previously tested as the minimum phosphate concentration allowing growth at 20 MPa (see material and methods for details). Growth was monitored every 9 h between *t* = 0 h and *t* = 105 h. Exponential phase was observed from 10 to 40 h at replete phosphate condition and from 10 to 50 h at reduced phosphate condition. Late exponential/stationary phase was observed from 40 to 105 h at replete phosphate conditions and from 50 to 105 h at reduced phosphate conditions (Fig. 1). Although the cultures at reduced and replete phosphate conditions were inoculated at different OD600 nm (Fig. 1), they reached the same OD_600nm_ during stationary phase.

### Changes in membrane lipids induced by change in pressure at replete and reduced phosphate concentration

‘*Labilibaculum euxinus*’ was grown in both replete (1.4 mM) and reduced (0.35 mM) phosphate (KH_2_PO_4_), both at 0.1 and 20 MPa, in triplicate, and harvested at *t* = 57 h corresponding to late exponential/early stationary phase (OD_600nm_ = 0.1, Fig. 1, see material and methods for details).

Phosphate-containing lipids (i.e. PEs and cardiolipins) and nonphosphate containing lipids (ornithine lipids, capnine lipids, glycine lipids, and flavolipins) were detected in all conditions. Capnine lipids were generally in the highest relative abundance (24%–33%), followed by ornithine lipids (25%–27%), PEs (24%–28%), cardiolipins (7%–13%), glycine lipids (8%–10%), and flavolipins (1%–2%) (Table 1, see Supplementary Table 1 for full list of lipids detected). It is important to note these relative abundances are based on peak area response and thus do not necessarily reflect their actual abundances due to differences in ionization efficiency. When ‘*L. euxinus*’ was previously grown at 0.1 and 20 MPa in rich medium (Yadav et al. 2020), we reported a somewhat different lipid distribution, with between 42% and 49% ornithine lipids, 24%–30% PEs, ∼10% capnine lipids, ∼4% flavolipins, and ∼1% glycine lipids. A previous study reported that, when grown in rich medium, *L. euxinus* contained ∼2%–4% PC lipids (Yadav et al. 2020), which were not observed in this study. Potentially their absence was due to the different culture conditions from the previous study or that they were in fact contamination from the rich medium used by Yadav et al. (2020). Cardiolipins were not reported in the Yadav et al. (2020) study, possibly as they were under the limit of detection or were not produced under those specific conditions.

**Table 1:** Distribution of IPL classes (average of *n*=3 and standard deviation) and degree of unsaturation of specific lipids detected in ‘*L. euxinus*’ A4^T^, under the four different conditions examined^a^.

	0.1 MPa		20 MPa	
	Replete PO_4_	Reduced PO_4_	Replete PO_4_	Reduced PO_4_
Ornithine lipids	27.0 ± 2.5	25.8 ± 3.0	25.8 ± 0.7	25.5 ± 1.4
Capnine lipids	26.4 ± 2.7	24.3 ± 4.0	28.8 ± 1.4	33.0 ± 8.0
Flavolipins	1.8 ± 0.1	2.4 ± 0.8	1.2 ± 0.2	1.6 ± 0.8
Glycine lipids	8.0 ± 0.4	10.4 ± 2.0	8.7 ± 1.0	8.8 ± 2.9
PE	23.6 ± 1.9	24.4 ± 0.8	28.1 ± 2.0	24.0 ± 5.2
Cardiolipins	13.2 ± 1.8	12.7 ± 0.6	7.3 ± 1.0	7.1 ± 0.6
[Unsaturated lipids]/[saturated lipids]^b^	0.15 ± 0.02	0.17 ± 0.01	0.29 ± 0.03	0.25 ± 0.05
[Unsaturated PEs]/[saturated PEs]^b^	0.9 ± 0.1	1.1 ± 0.1	2.8 ± 0.7	3.5 ± 1.6

^a^It should be noted that IPLs have varying degrees of ionization efficiency and hence the peak areas of different IPLs, used to calculate these percentages, do not necessarily reflect their actual relative abundance. ^b^Sum does not include cardiolipins.

In the present study, the most noticeable lipid adaptation between low and high pressure was the change in the relative amount of cardiolipins (Table 1). In the two sets of triplicate cultures grown at 0.1 MPa, that were grown with replete phosphate contained 13.2 ± 1.8% cardiolipins, while those grown at reduced phosphate contained 12.7 ± 0.6% cardiolipins. This number was reduced in the two sets of triplicate cultures grown at 20 MPa, to a very similar degree. At that pressure, cardiolipins made up 7.3 ± 1.0% at replete phosphate and 7.1 ± 0.6% at reduced phosphate. What was different between the replete and reduced phosphate cultures, was which lipid class concomitantly increased, as cardiolipins decreased. The cultures grown with replete phosphate saw an increase in the phospholipid PE (from 23.6 ± 1.9% at 0.1 MPa to 28.1 ± 2.0% at 20 MPa) while the cultures grown at reduced phosphate experienced an increase in the amino lipid capnine (from 24.3 ± 4.0% at 0.1 MPa to 33.0 ± 8.0% at 20 MPa; which in this case was not significant, see Supplementary Table 2).

The ratio of unsaturated/saturated phospholipids (not including cardiolipins; Table 1 and Supplementary Table 1), was higher with increased hydrostatic pressure (with ratios of 0.15 ± 0.02 and 0.17 ± 0.01 at atmospheric pressure and ratios of 0.29 ± 0.03 and 0.25 ± 0.05 at 20 MPa, at replete and reduced phosphate concentrations, respectively). This was more noticeable within the PEs, where the unsaturated/saturated ratio increased from 0.9 ± 0.1 and 1.1 ± 0.1 at 0.1 MPa to 2.8 ± 0.7 and 3.5 ± 1.6 at 20 MPa, at replete and reduced phosphate concentrations, respectively (Table 1 and Supplementary Table 1).

### Morphological changes induced by changes in phosphate availability and HHP

**Figure 2:**
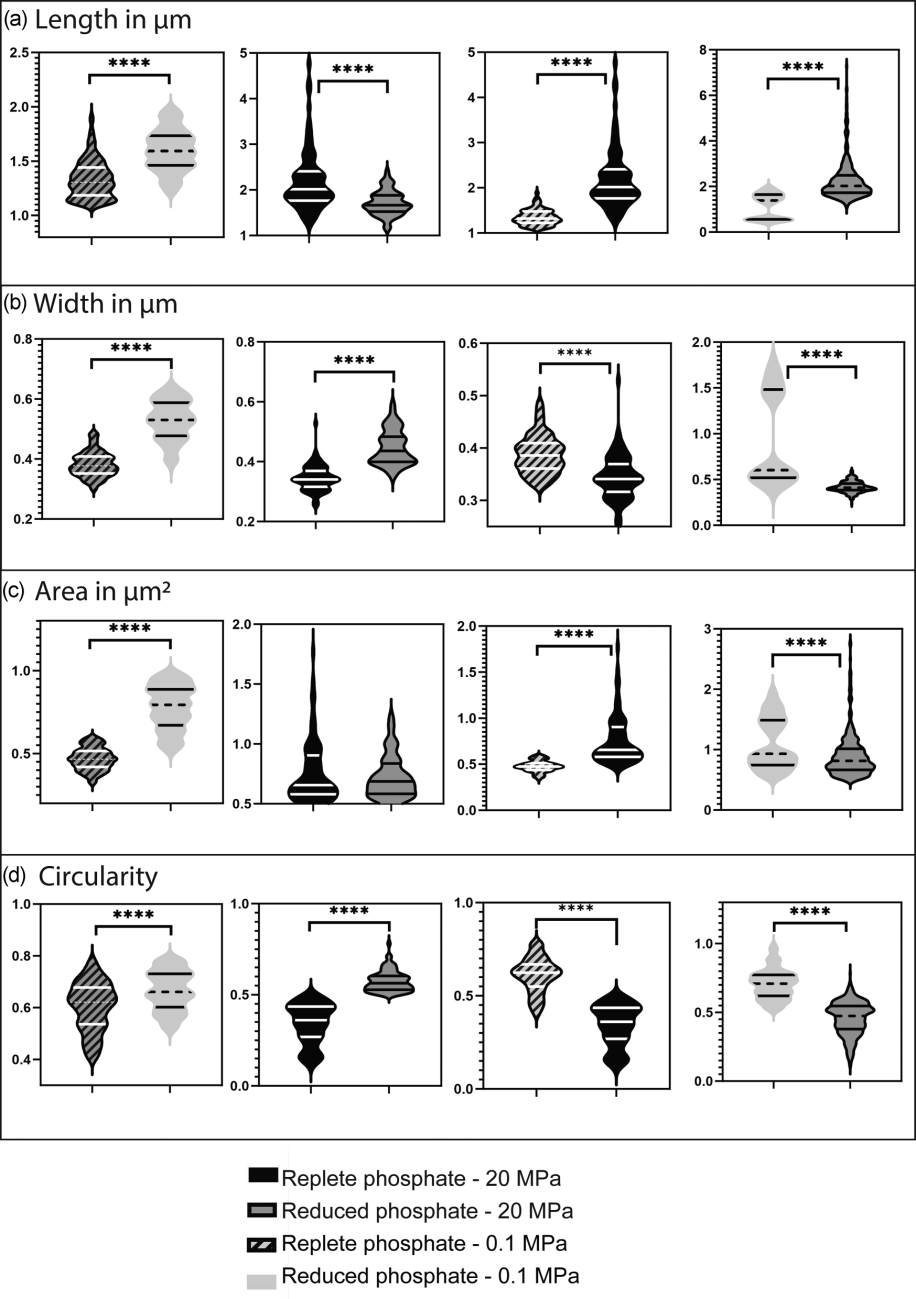
Morphological changes observed in ‘*L. euxinus*’ summarized in four parameters and plotted as violin charts (a) length in µm, (b) width in µm, (c) area in µm^2^, and (d) circularity. *** and **** means *P* ≤ .001 and *P* ≤ .0001, respectively.

We evaluated changes in the cell shape and membrane properties of ‘*L. euxinus*’ under each of the conditions tested. At atmospheric pressure (0.1 MPa), ‘*L. euxinus*’ had a rod shape of between 1 and 1.5 µm length and a width between 0.4 and 0.5 µm. Increased hydrostatic pressure induced an elongation of the cells, with the length doubling from 1.3 to 2.2 µm (±0.6 µm) at replete phosphate conditions, and from 1.1 to 2.2 µm (±0.2 µm) at reduced phosphate conditions (Fig. 2a). The same trend was observed when the cells were exposed to different phosphate concentrations at atmospheric pressure, with cell length increasing from 1.3 to 1.5 µm (±0.5 µm) when phosphate concentration decreased. However, the opposite trend was observed under HHP, with cell length decreasing from 2.2 to 1.7 µm (±0.2 µm) with decreasing phosphate concentration (Fig. 2a).

We examined the influence of phosphate availability and HHP on cell width, revealing significant effects across all tested conditions (Fig. 2b). In both replete and reduced phosphate concentrations, cells were wider at atmospheric pressure than at 20 MPa. The width at atmospheric pressure at replete phosphate was 0.38 µm, and 0.5 µm at reduced phosphate condition. Likewise, cells were wider at reduced phosphate concentration at both pressures tested, with width of 0.52 µm at 0.1 MPa and 0.44 µm at 20 MPa (Fig. 2b).

We observed variations in cell area (see Material and Methods for details). The increase in pressure affected the cells differently depending on phosphate concentration. At replete phosphate concentration, increased pressure induced an increase of cell area from 0.47 to 0.75 µm^2^ while at reduced phosphate concentration, the opposite trend was observed with a decrease from 1 to 0.89 µm^2^ (Fig. 2c). At atmospheric pressure (0.1 MPa), cell area almost doubled from 0.45 to 0.79 µm^2^ with decreased phosphate concentrations, while at 20 MPa, cells remained of the same area regardless of phosphate concentrations, ~0.75 µm^2^ (Fig. 2c).

Finally, cell shape roundness was assessed through circularity measurements, as indicated in the Material and Methods. Significant changes were observed across different pressure and phosphate conditions. Between 0.1 and 20 MPa, there was a notable decrease in circularity at HHP, which was amplified by lower phosphate concentration, with a decrease from 0.61 to 0.34 in replete phosphate condition and a decrease from 0.7 to 0.45 at depleted phosphate concentration (Fig. 2d). Conversely, changes in phosphate concentration also produced significant effects. Specifically, between 1.4 and 0.35 mM of phosphate, circularity increased as phosphate concentration decreased with an accentuated effect at HHP, with an increase from 0.6 to 0.66 at 0.1 MPa and an increase from 0.34 to 0.57 at 20 MPa (Fig. 2d).

### Genomic potential for amino lipid biosynthesis in ‘*L. euxinus*’

‘*Labilibaculum euxinus*’ has been seen to produce a high relative abundance of amino lipids (~65%) in this study. Several amino lipid biosynthetic pathways have been described in the recent years (Liu et al. 2024), especially for the case of ornithine lipid biosynthesis, indicating a wide taxonomic and functional diversity of amino lipid biosynthesis within Bacteria (Vences-Guzmán et al. 2012, 2015, Rivera-Najera et al. 2025), which justifies an in deep genomic analysis of biosynthetic potential in amino lipid strain producers. To this end, here we determined the genomic potential for amino lipid biosynthesis in ‘*L. euxinus*’ to further clarify the biosynthetic pathways leading to the synthesis of those amino lipids. Here, we screened the genome of ‘*L. euxinus*’ for homologs of the biosynthetic genes coding for the different enzymes involved in the synthesis of amino lipids, including those involved in the olsB/A pathway (Vences-Guzmán et al. 2012) present mainly in species of the phylum *Pseudomonadota*, as well as in some Gram-positive bacteria with the *Mycobacterium* and *Streptomyces* genera (Geiger et al. 2010, Sohlenkamp and Geiger 2015); and the key coding gene of the OlsF pathway (Vences-Guzmán et al. 2015), found in some bacteria of the FCB group (see Supplementary Fig. 2). No homologs of OlsA/B were detected in the genome of *L. euxinus*. Nevertheless, a potential homologue of the OlsF coding gene was detected in the genome (Table 2), which agrees with the synthesis of ornithine lipids using OlsF, as already observed in other members of the FCB group (Vences-Guzmán et al. 2015). Homologs with quite high support (high % of identity and low *e*-value, see Table 2 for details) to the previously confirmed genes involved in the synthesis of the amino lipids glycine lipids (i.e. GlsAB) and of capnine lipids (CapABC), were also detected (Table 2; Supplementary Fig. 2).

**Table 2:** Homology of the confirmed proteins involved in ornithine, glycine and capnine lipid biosynthesis, with the annotated genome of ‘*L. euxinus*’ A4^T^ obtained by tblastn in NCBI.

	Ornithine lipid biosynthesis			Glycine lipid biosynthesis		Capnine lipid biosynthesis		
Name query protein	OlsA	OlsB	OlsF	GlsB	GlsA	CapA	CapB	CapC
% identity	28%	25%	32%	57%	47%	32%	61%	37%
*e*-value	2e^−7^	0.001	7.2e^−58^	7.6e^−115^	1.9e^−54^	1.1e^−42^	3.4e^−138^	9.5e^−35^
Location genome	4 413 479–4 413 192	106 330–105 761	2 946 711–2 945 866	106 597–105 623	107 568–107 029	2 068 032–2 068 916	3 601 233–3 602 204	4 304 459–4 305 178

GlsA, O-acyltransferase (BT_3458*, Bacteroides thetaiotaomicron* VPI-5482, accession number NCBI AAO78564.1, Phospholipid/glycerol acyltransferase); GlsB, N-acyltransferase (BT_3459, *Bacteroides thetaiotaomicron* VPI-5482, accession number (AAO78565.1, annotated as a hemolysin A) (Lynch et al. 2019), OlsA *Sinorhizobium meliloti* (AEH77326.1), OlsB *S. meliloti* (ATA97800.1), OlsF *Serratia proteamaculans* (QQX52868.1) (Vences-Guzmán et al. 2015); CapA (Uniprot C7M8J5, NCBI accession ACU93374.1 *Capnocytophaga ochracea*), CapB (C7M8J6; ACU93375.1, *Capnocytophaga ochracea*), CapC (A0A3R5XUE6, QAR30703.1, *Ornithobacterium rhinotracheale*) (Liu et al. 2022).

## Discussion

This study aimed to further constrain the adaptation mechanisms involving membrane lipids in the marine piezotolerant ‘*L. euxinus*’ to changes in phosphate concentrations and hydrostatic pressure. The strain of ‘*L. euxinus*’ (Yadav et al. 2020) was grown in the current study in a defined media with pyruvate as carbon source and different phosphate concentrations, being the minimum concentration of phosphate able to sustain growth 0.35 mM phosphate (i.e. referred here as ‘reduced phosphate’) at 20 MPa, and the maximum used is this study was 1.4 mM phosphate, corresponding to the phosphate concentration in the rich medium originally used for the isolation of *L. euxinus* (Yadav et al. 2020; referred here as ‘P replete’). The concentration of dissolved phosphate in the deep waters of the Black Sea, where this strain was originally isolated, was 8 µM (Suominen et al. 2022), which is substantially lower than the minimum concentration of phosphate sustaining growth in culture at 20 MPa. The differences between the minimum phosphate concentration for growth in culture and the *in situ* concentration in the environment where the strain was isolated can be explained by (i) an extra supply of phosphate from microorganisms present in the same habitat in which ‘*L. euxinus*’ was isolated (Dijkstra et al. 2018), (ii) from iron-phosphate minerals accumulated by sulfur-disproportionating Deltaproteobacteria known to be present in the deep euxinic waters of the Black Sea (Dijkstra et al. 2014, Suominen et al. 2022), and/or (iii) from the culture-mode bias, as batch culture selects for fast, high-yield growth inflating apparent phosphate requirements (Norris et al. 2021) versus slow and low P-requirement *in situ* growth in the environment in which the strain was isolated.

Morphological changes during the growth phase suggest that ‘*L. euxinus*’ had to adapt to the changing conditions, i.e. rounder cells, which are generally less efficient at nutrient uptake but gain advantages in energy conservation (Young 2007).

Morphological changes in ‘*L. euxinus*’ induced by changes of hydrostatic pressure or nutrient concentration would suggest that its membrane lipid composition was impacted by growth conditions (Ramos-Martín and D’Amelio 2022). We observed differences in the membrane lipid composition of ‘*L. euxinus*’ grown in defined media with pyruvate as carbon source for this study in comparison with the membrane composition when grown in rich media containing yeast extract and peptone (Yadav et al. 2020). Notably, the differences of relative abundance of capnine lipids, ornithine lipids and glycine lipids suggest that *L. euxinus* modifies its membrane composition depending on carbon source/availability. This difference could be justified by changes in nutrient availability, redox potential or polarity (Ramos-Martín and D’Amelio 2022). Alternatively, differences detected here in comparison with the previous study of Yadav et al. (2020) could be potentially explained by the fact that the strain had been adapted longer (~100 transfers) to the defined media in our experiment in comparison with the rich medium used by Yadav et al. (2020), and potentially changed its IPL composition in successive transfers.

‘*Labilibaculum euxinus*’ kept a relatively constant distribution of IPL classes (ornithine lipids, capnine lipids, PE, glycine lipids, flavolipins, and cardiolipins) across both phosphate concentrations, despite the changes in cell morphology and extended lag phase in growth at reduced phosphate concentration. This suggests that ‘*L. euxinus*’ has an unusually stable membrane composition which can withstand changes of environmental parameters with a minimum change in its lipid distribution. This resilience of ‘*L. euxinus*’ is unusual. For example, phosphate limitation has been identified to influence the polar headgroup distribution in bacterial groups, such as in *Rhizobium tropici* and in genetically engineered strains of *E. coli* (Vences-Guzmán et al. 2011, Winnikoff et al. 2024). For example, phosphate limitation has been seen to increase the relative abundance of In *Sinorhizobium meliloti* and in *Desulfatibacillum alkenivorans, phosphate limitation induces an increase in* nonphosphate containing lipids, such as ornithine lipids, and betaine lipids, respectively (Lopez-Lara et al. 2007, Vences-Guzmán et al. 2015, Bosak et al. 2016, Ding et al. 2024). Our defined medium contained phosphate (as KH_2_PO_4_) with a concentration much higher than the one *in situ* in the Black Sea, it would not be expected that ‘*L. euxinus*’ was challenged in its growth. On the other hand, the reduced phosphate concentration used in our study was the lowest concentration that could still sustain growth at 20 MPa. Nevertheless, ‘*L. euxinus*’ grown in ‘reduced’ phosphate conditions reached the same OD_600nm_ than the strain growth in ‘replete’ phosphate conditions, suggesting that the strain is not P-limited under the conditions tested. Despite of the fact that P-limitation is not expected under the selected growth conditions, the cell morphology changed upon phosphate reduction, while IPLs did not.

In addition to changes of phosphate concentrations, ‘*L. euxinus*’ was challenged to grow at two different hydrostatic pressures. Pronounced morphological changes were also observed between 0.1 MPa and 20 MPa, both at replete and reduced phosphate concentration, with elongated cells being observed at 20 MPa in both cases, which could be associated with response to stress, or resource efficiency uptake, suggesting that ‘*L. euxinus*’ can tolerate an increase of hydrostatic pressure but with a tradeoff in its fitness. Despite the observed changes in the growth, the distribution of lipids classes remained relatively constant during growth at 0.1 and 20 MPa, further indicating that the lipid membrane of ‘*L. euxinus*’ can ‘buffer’ considerable environmental changes, without undergoing significant membrane lipid remodeling.

One notable change in the membrane composition associated with pressure, was the decrease in the relative percentage of cardiolipins at 20 MPa, relative to 0.1 MPa. To our knowledge, this is the first evidence suggesting that cardiolipins play a role in membrane adaptation to HHP. Cardiolipins, also known as 1,3-bis (sn-3′-phosphatidyl)-*sn*-glycerols or bisphosphatidylglycerols, are a class of phospholipids or dimeric phospholipids in which two 1,2-diacyl-sn-glycero-3-phosphoryl moieties are bound through a third glycerol moiety (see Supplementary Fig. 1 for structures). They are anionic phospholipids with two negatively charged phosphatidic moieties attached to four hydrophobic acyl chain through a glycerol backbone and increase in their abundance has been shown to increase fluidity in the lipid bilayer (Unsay et al. 2013). Additionally, cardiolipins contribute to the stabilization and binding of membrane proteins, a feature that may contribute to maintaining membrane integrity under HHP (Lewis and McElhaney 2009, Musatov and Sedlák 2017). Moreover, due to their high negative charge, cardiolipins are implicated in transport processes, potentially facilitating the uptake of essential nutrients required for survival under HHP (Musatov and Sedlák 2017). Cardiolipins have also been shown to help anchor and stabilize cytochrome c oxidases in respiratory chains, thus optimizing electron transport and supporting energy production (Musatov and Sedlák 2017). In *Shewanella violacea*, a piezophilic bacterium, cytochrome *c* has been shown to play a role in pressure-adaptation—although the molecular mechanism remains unknown (Yamada et al. 2000). We hypothesize that a similar adaptation might occur in ‘*L. euxinus*’, namely an increase in cardiolipins helps overcome the effect of HHP by acting as anchors for membrane proteins, e.g. cytochrome *c*, preventing dissociation. Cardiolipins have also a conical shape (small headgroup, bulky acyl chains), which influences membrane curvature and morphology in general (e.g. Wood 2018, Lin et al. 2019), by localizing in highly curved regions (e.g. cell poles). At HHP, cardiolipins would be expected to create functional microdomains in the cell poles but likely does not counteract the effect of HHP of compressing and elongating cells, like we have observed in ‘*L. euxinus*’.

Another pressure-induced membrane lipid change detected in ‘*L. euxinus*’ was increased lipid unsaturation, a strategy commonly found in piezotolerant microorganisms. For example, several studies have observed an increase in the unsaturation of the phospholipid fatty acids under HHP (Bartlett 1999, Allen et al. 1999), including the role of the fatty acid unsaturation in the stabilization of the cellular membrane under HHP (e.g. Usui et al. 2012). In ‘*L. euxinus*’ this increase of unsaturation affected lipids with a PE headgroup, regardless of phosphate concentration. Overall, the changes to the ‘*L. euxinus*’ membrane lipid composition under changes of phosphate concentration and hydrostatic pressure were relatively minor, which suggests that ‘*L. euxinus*’ has a harmoniously optimized membrane composition, which can efficiently adapt to multiple challenges. The high abundance of nonphosphate containing headgroups, i.e. amino lipids, regardless of the tested conditions, suggests that the nature of these lipids might be responsible for the membrane robustness of this strain.

In recent years, several amino lipid biosynthetic pathways have been identified and characterized. In some cases, such as ornithine lipid biosynthesis, this has revealed that these pathways are both taxonomically widespread and functionally diverse. In this case, we screened the genome of ‘*L. euxinus*’ to assess the potential pathways involved in the amino lipids detected in our study. ‘*Labilibaculum euxinus*’ membrane contains a high proportion of ornithine lipids (Table 1). Associated with this lipid, the biosynthetic genes *olsF* was found, as to be expected in bacteria from the FCB group. Ornithine lipids are mostly associated with phosphate limitations; however, their relative abundance has remained consistently high, regardless of the growth media, which suggests that the high relative abundance of ornithine lipids, and of amino lipids in general, in FCB strains could respond to other reasons. Ornithine lipids have been identified to play an important structural role in *Desulfovibrio gigas* (phylum *Thermodesulfobacteriota*), and to increase antibiotic susceptibility in *Pseudomonas aeruginosa* (phylum *Pseudomonadota*; Makula and Finnerty 1975, Kim et al. 2018). Considering ‘*L. euxinus*’ physiology, it is likely its main use for ornithine lipids is structural—however further investigation into cell interactions and signaling could provide further insight into its role.

Another major component of ‘*L. euxinus*’ are capnine lipids, in about the same proportion as ornithine lipids. Behind the biosynthesis of this lipid, the genes *capABC* were found in *L. euxinus* genome. Capnine lipids are a class of sulfonolipids built on a capnine backbone, a long-chain sulfonic acid that replaces the typical fatty acid carbonyl group with a sulfonic acid group. Capnine lipids are thought to play a role in the motility of gliding Bacteroidetes (Godchaux III and Leadbetter 1984). Capnine lipids are synthesized from cysteate, which has been shown to compensate for gliding and sulfolipid biosynthesis impairment (Liu et al. 2022). Although the capacity of gliding has not been assessed in *L. euxinus*, the presence of capnine lipids could be related with a potential gliding capacity which could explain this strain resilience to the wide range of tested conditions. Follow-up studies would need to assess this capacity and its potential adaptive strategy.

Genes coding for the enzymes involved in the glycine lipid biosynthetic were also found in the ‘*L. euxinus*’ genome (i.e. *glsAB*, Table 2). Those genes were first identified in *Bacteroides thetaiotaomicron*, also a member of the FCB clade, a beneficial bacterium found in human gut microbiota (Lynch et al. 2019). In *B. thetaiotaomicron*, glycine lipids have been identified to play an important role in resistance to oxygen exposure (Lynch et al. 2019). Considering that ‘*L. euxinus*’ is a facultative anaerobe, it is possible that glycine lipids play a similar role in this organism, pending further investigation.

## Conclusion

Our findings demonstrate that ‘*L. euxinus*’ exhibits noticeable membrane resilience when exposed to varying phosphate concentrations and hydrostatic pressures. Despite the tested environmental stressors, this piezotolerant microorganism maintained a consistent distribution of membrane lipid classes, with a notable dominance of amino acid–containing lipids such as ornithine, capnine, and glycine lipids. These results suggest an inherent structural robustness potentially linked to the organism’s stable lipidome. While morphological changes, such as elongation under high pressure, indicate physiological responses, IPL composition shifts were minimal, aside from increased unsaturation and reduced cardiolipin content at elevated pressures.

We recognize that the physiological patterns identified under the experimental conditions tested here cannot be assumed to mirror those occurring in the deep Black Sea. The phosphate concentrations required for sustained growth (0.35 mM) exceed natural levels by more than an order of magnitude, and the use of 28°C rather than ~8°C *in situ* temperature in the deep Black Sea water column, likely promotes a metabolic activity not representative of environmental growth. While these parameters enabled us to quantify membrane lipid responses under defined conditions, they may diverge from the organism’s ecological state *in situ*. Thus, the ecological relevance of our findings should be interpreted with caution, and future work incorporating more environmentally realistic nutrient and thermal conditions will be necessary to resolve this gap. Nevertheless, the controlled approach if the current study still provides a valuable framework for disentangling specific physiological and lipidomic responses to pressure and nutrient availability, offering mechanistic insights that are difficult to obtain directly from complex environmental samples.

In addition, genomic analysis supported the biosynthetic capacity for key membrane amino lipids, reinforcing their functional importance. This study underscores the limited plasticity yet high efficiency of ‘*L. euxinus*’ membrane adaptation and highlights the need for further exploration into how lipid composition contributes to environmental tolerance in extremophiles.

## Supplementary Material

fiag012_Supplemental_Files

## Data Availability

‘*Labilibaculum euxinus*’ genome, lipid sequences and microscopy are available at 10.5281/zenodo.14721972.
