# Supplementary material for: Exceptionally stable membrane lipid composition of the marine facultative anaerobe and piezotolerant ‘Labilibaculum euxinus’ under variable pressure and nutrients
Source: FEMS Microbiol Ecol. 2026 Feb 18;102(3):fiag012. doi: 10.1093/femsec/fiag012 (PMC12927429; doi:10.1093/femsec/fiag012)

# **Supplementary Information**

Exceptionally stable membrane lipid composition of the marine facultative anaerobe and piezotolerant ‘Labilibaculum euxinus’ under variable pressure and nutrients

Anandi Tamby^1^, Diana X. Sahonero-Canavesi^1^, Nicole J. Bale^1^, Laura Villanueva^1,2^

^1^Department of Marine Microbiology and Biogeochemistry (MMB), NIOZ Royal Netherlands Institute for Sea Research, Den Burg, Netherlands.

^2^Department of Biology, Faculty of Sciences, Utrecht University, Utrecht, Netherlands.

# ***Corresponding author**: laura.villanueva@nioz.nl

**Supplementary Figure 1** – The structure of the intact polar lipids detected in *L. euxinus*: ornithine lipid, capnine lipid, glycine lipid, flavolipin, phosphatidylethanolamine (PE) and cardiolipin.


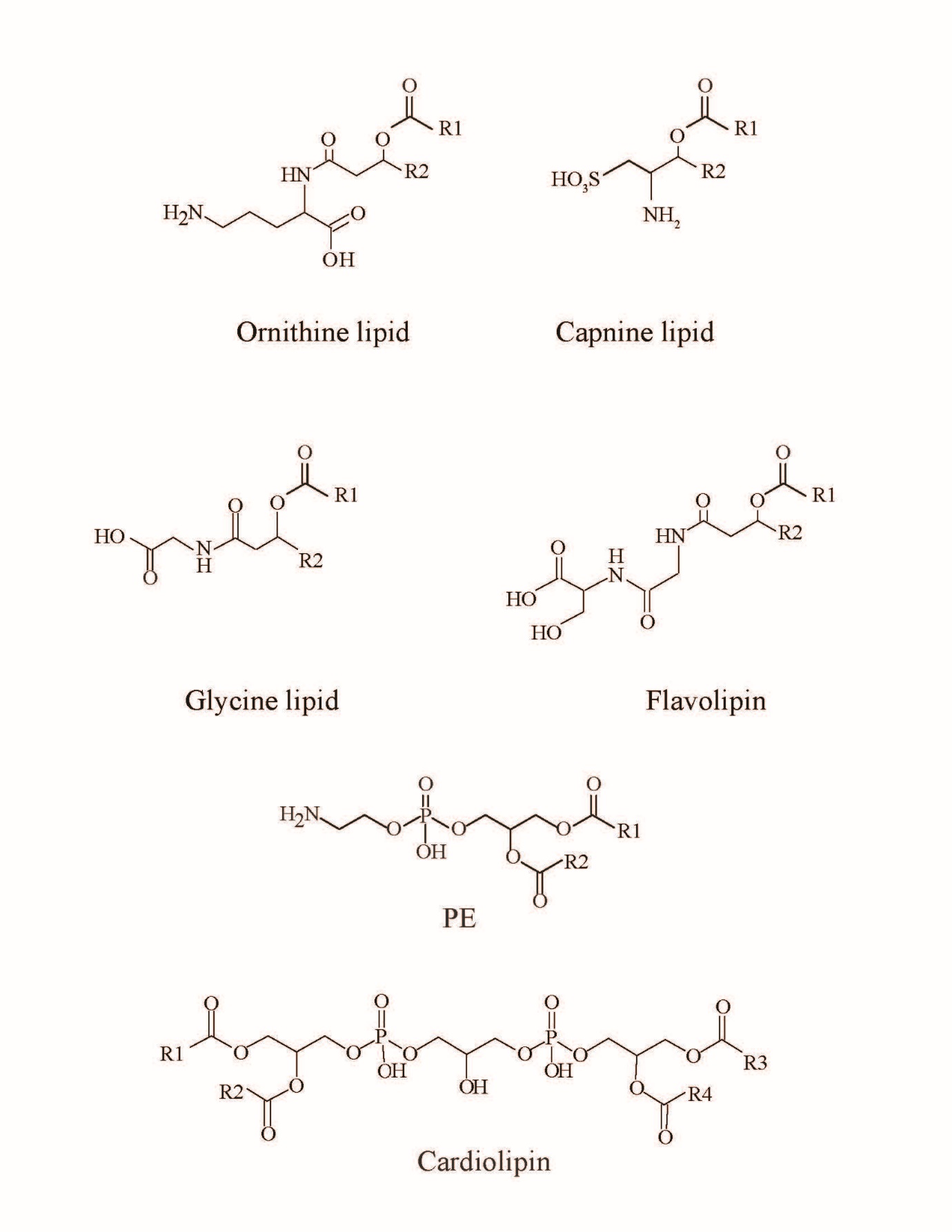


**Supplementary Figure 2** – Lipid biosynthetic pathways investigated. A) glycine lipids biosynthesis with GlsB, known to be a N-acyltransferase involved in the formation of glycine lipids (Lynch et al. (2019); B) capnine lipid biosynthesis, with CapA catalyzing the formation of cysteate from *O*-phospho-l-serine and sulfite, followed by CapB catalyzing the formation of dehydrocapnine from cysteate and 13-methyl-myristoyl-CoA, and the reduction of 3-dehydrocapnine by CapC into capnine. C) ornithine lipid biosynthesis, the olsB/A pathway consisting of a *N-*acyltransferase forming the amide bond leading to lyso-OL from ornithine, followed by an *O*-acyltransferase forming the ester bond between lyso-OL and acyl-AcpP forming OL and the OlsF pathway which consist of one single protein coded by the *olsF* gene, with the C-terminal domain of OlsF being responsible for the N-acyltransferase activity and the N-terminal domain of OlsF being responsible for the *O*-acyltransferase reaction (Vences-Guzmán et al., 2012, 2015).


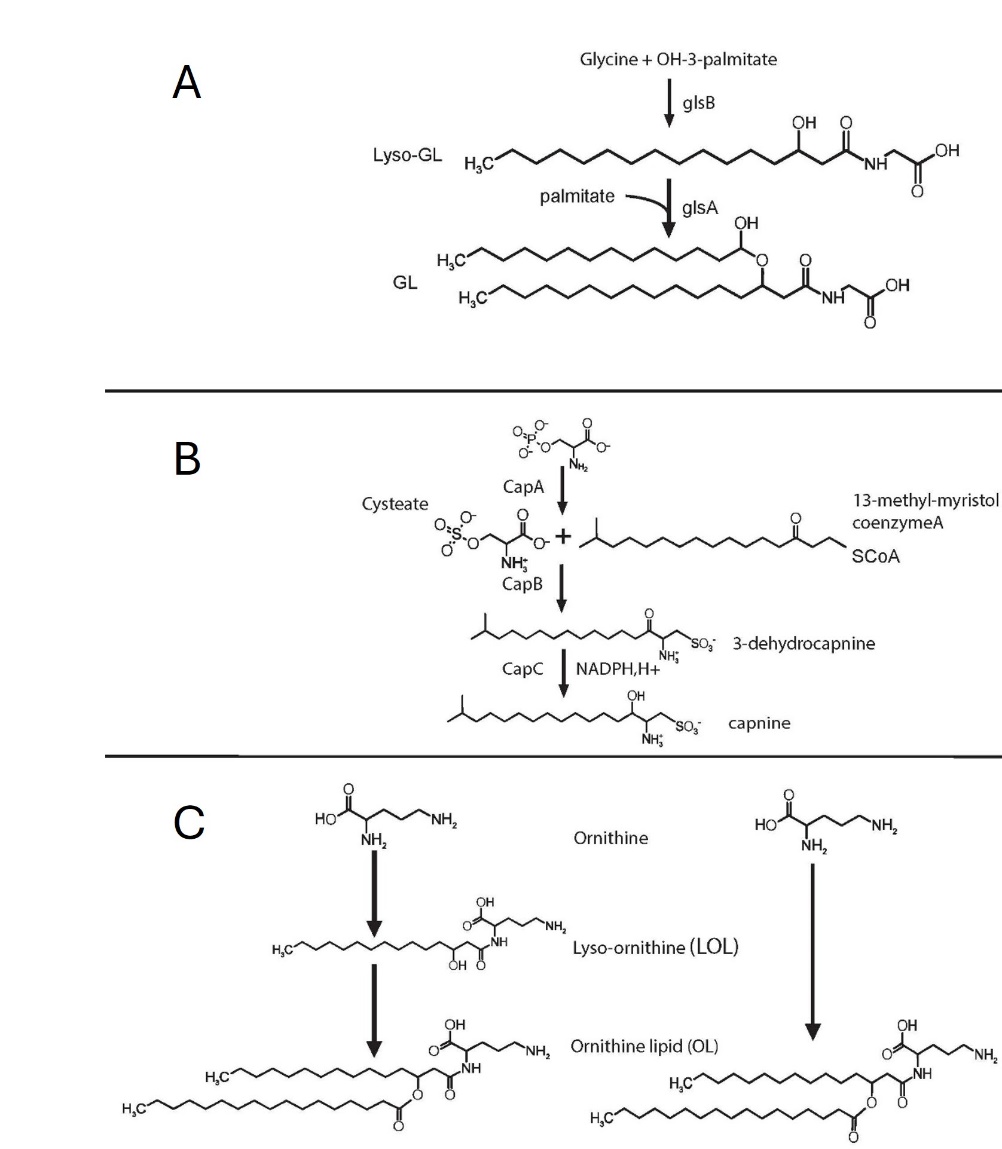

Supplement: fiag012_Supplemental_Files [file fiag012_supplemental_files.zip › Supplementary data_villanueva_version20.docx]
